# The inclusion of mental health in the international public health agenda and the leading role of nursing in this process

**DOI:** 10.1590/1518-8345.0000.3012

**Published:** 2018-06-18

**Authors:** Sueli Frari Galera

**Affiliations:** Doctor in Nursing, Associate Professor 2, Escola de Enfermagem de Ribeirão Preto, Universidade de São Paulo, PAHO/WHO Collaborating Centre for Nursing Research Development, Brazil. E-mail: sugalera@eerp.usp.br

**Figure:**
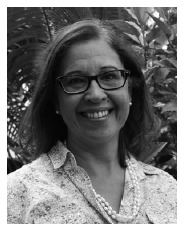

Primary health care as a key element in meeting the health needs of the populations across the world has been generally highlighted in the nursing and health literature. In this context, it is worth emphasizing mental health as an integral part of primary health care in its global perspective.

Although the concept of health brings the idea of ​​mental health, since it represents not only the absence of diseases, but also the physical, psychic and social welfare, mental health is often relegated to a smaller focus of the discussions about health care. Nonetheless, the global situation of uncertainties with global challenges such as violence, terrorism, climate change, displacements of individuals and families, has demanded the inclusion of mental health in the international public health agenda.

Seen in these terms, one should highlight how mental health was present in all subjects discussed during the Congress of the International Council of Nurses (ICN) held in Spain in 2017 ^1^.

In the subject of “Strengthening health systems”, a special group of works focused on the care for the heart and mind highlighted the role of mental health in this issue. Another subject was “Sustainability of health care, disasters and conflicts, with expositions targeted towards the mental health needs in these contexts. Commonly, this subject does not address mental health issues because the main focus is on physical interventions to save lives. Nonetheless, nurses understand the long-term sequels intertwined with these circumstances.

Expositions focused on strengthening the preparation of mental health specialists and general practitioners to care for individuals of all ages, in different care environments, with all care focused on the quality and safety of care, were also present in the works exposed. In the subject of “Innovations in professional practice and in health policies”, papers on innovative interventions were exposed, including health equity for those individuals who are often marginalized by our society, for example, mentally disabled persons and users of illegal substances.

Although mental health nurses have been showing promising evidence on interventions, research and teaching, there is a challenging gap in this field that is the existence of few studies on the translation of evidence-based practice into the scope of mental health care and psychiatric treatment ^2^. This gap has been signalized with regard to family inclusion ^(^ ^3^ ^)^ and innovations in the care of children and adolescents with mental health problems ^2^. Researchers in this field assert that this lack of disclosure hampers this field and makes it difficult for customers and their families to access evidence-based approaches.

Around the world, health systems deal with challenges to improve the quality of care, and seek to reduce the discrepancy between knowledge produced and practice, in addition to identifying facilitators in this process. The learning about how to apply evidence of what is already known, how to identify, prioritize and find relevant research to produce new knowledge is a challenge in many areas of nursing ^4^.

Given the fact that nurses are the majority of the labor power of many health services, they can make an important contribution in this translational work and its disclosure ^1^ ^,^ ^2^. Nurses have more contact with customers and their families than any other professional of the interdisciplinary team. This contact enables a single point of view on the perspective of those who need help before the dilemma they face ^2^.

Accordingly, nurses are in an excellent position to take the lead in evidence-based practice because the tools to follow-up the translation are accessible to the nursing work, which is at the forefront of the care provided by the multidisciplinary team. Nurses can make a difference, not only in the direct patient care, but also in the development and implementation of policies. The influence of nurses on mental health is promising for the future of our nursing work in our global societies. 
